# The Role of Deoxycytidine Kinase (dCK) in Radiation-Induced Cell Death

**DOI:** 10.3390/ijms17111939

**Published:** 2016-11-21

**Authors:** Rui Zhong, Rui Xin, Zongyan Chen, Nan Liang, Yang Liu, Shumei Ma, Xiaodong Liu

**Affiliations:** 1 Key Laboratory of Radiobiology (Ministry of Health), School of Public Health, Jilin University, Changchun 130021, China; rui.zhong1702@gmail.com (R.Z.); xin-1017@163.com (R.X.); czy081206@163.com (Z.C.); zrcc111@126.com (N.L.); lyy2405@126.com (Y.L.); 2 Department Radiology, the 2st Hospitals Affiliated to Jilin University, Changchun 130021, China; 3 Department Diagnostic Imaging, Weihai Chest Hospital, Weihai 264220, China; 4 Department Radiation Oncology, the 2nd Hospitals Affiliated to Jilin University, Changchun 130021, China

**Keywords:** deoxycytidine kinase (dCK), autophagy, apoptosis, mitotic catastrophe, radiation

## Abstract

Deoxycytidine kinase (dCK) is a key enzyme in deoxyribonucleoside salvage and the anti-tumor activity for many nucleoside analogs. dCK is activated in response to ionizing radiation (IR)-induced DNA damage and it is phosphorylated on Serine 74 by the Ataxia-Telangiectasia Mutated (ATM) kinase in order to activate the cell cycle G2/M checkpoint. However, whether dCK plays a role in radiation-induced cell death is less clear. In this study, we genetically modified dCK expression by knocking down or expressing a WT (wild-type), S74A (abrogates phosphorylation) and S74E (mimics phosphorylation) of dCK. We found that dCK could decrease IR-induced total cell death and apoptosis. Moreover, dCK increased IR-induced autophagy and dCK-S74 is required for it. Western blotting showed that the ratio of phospho-Akt/Akt, phospho-mTOR/mTOR, phospho-P70S6K/P70S6K significantly decreased in dCK-WT and dCK-S74E cells than that in dCK-S74A cells following IR treatment. Reciprocal experiment by co-immunoprecipitation showed that mTOR can interact with wild-type dCK. IR increased polyploidy and decreased G2/M arrest in dCK knock-down cells as compared with control cells. Taken together, phosphorylated and activated dCK can inhibit IR-induced cell death including apoptosis and mitotic catastrophe, and promote IR-induced autophagy through PI3K/Akt/mTOR pathway.

## 1. Introduction

Cell death is a fundamental biological process that has been mediated via intracellular program of biological systems [1,2]. The execution of cell death needs an orchestrated interplay between some important processes: apoptosis, autophagy, necrosis [3] and mitotic catastrophe [4]. Apoptosis refers to a constellation of characteristic changes leading directly to cell death [5]. Over the past few decades, apoptosis has been widely studied, and radiotherapy strategies targeting apoptosis has become one of the most important cancer treatments [6]. Autophagy is a kind of catabolic process for maintaining cellular homeostasis and supplying substrates for energy generation [7]. Under stresses, however, autophagy may protect cells or lead to cell death [8]. Therefore, autophagy is considered a double-edged sword in the process of tumor development.

Other than apoptosis and autophagy, mitotic catastrophe is one of the atypical deaths defined by the Nomenclature Committee on Cell Death (NCCD) [9,10]; it also acts as an oncosuppressive mechanism which can prevent perturbations of the mitotic machinery and genomic instability in response to DNA damage [11]. A consequence of failure to complete mitosis and accumulation of unrepaired DNA damage can lead to mitotic catastrophe [4]. The G2 phase checkpoint is important for preventing mitotic cell death. A transient cell cycle will not let cells have enough to repair DNA and then proceed into mitosis, fail to separate, leading to catastrophic cell division [12].

Deoxycytidine kinase (dCK) is a key enzyme in the deoxyribonucleoside salvage, which is important to maintain normal DNA metabolism. dCK can activate many antiviral and anticancer nucleoside analogs, such as fludarabine, gemcitabine, cladribine and zalcitabine [13]. dCK can also be responsible for the phosphorylation of deoxycytidine (dC), deoxyadenosine (dA), and deoxyguanosine (dG) to their monophosphate forms [14] and is also involved in DNA damage repair [15,16]. dCK protein has four phosphorylation sites, Thr-3, Ser-11, Ser-15, Ser-74 [17]. dCK activity can be increased by Ser-74 phosphorylation [18] and Thr-3 can promote the stability of dCK [17]. Radiation and genotoxic drugs such as aphidicolin, etoposide and certain nucleoside analogs can increase dCK kinase activity [19,20]. dCK can activate many nucleoside analogs that have exhibited synergistic activity with radiotherapy to inhibit DNA repair [21]. Moreover, dCK deficiencies or dCK mutants (G12) can mediate resistance to nucleoside analogs [22,23]. In addition, dCK is used as a biomarker and drug target in clinical application [24].

Our previous studies have demonstrated that Ataxia-telangiectasia-mutated (ATM) kinase can phosphorylate dCK on Serine 74 to activate it in response to ionizing radiation (IR) treatment and dCK regulates the G2/M checkpoint through interaction with cyclin-dependent kinase 1 in response to DNA damage [25]. Ataxia-Telangiectasia Mutated (ATM) could promote IR-induced autophagy via the Akt/mTOR/P70S6K pathway [26]. In this study, we focused on further characterization of dCK phosphorylation on S74 in radiation-induced cell death and its possible mechanism. This will provide a new insight for targeting therapy of cancer.

## 2. Results

### 2.1. Deoxycytidine Kinase Decreased Radiation-Induced Cell Death

**Figure 1:**
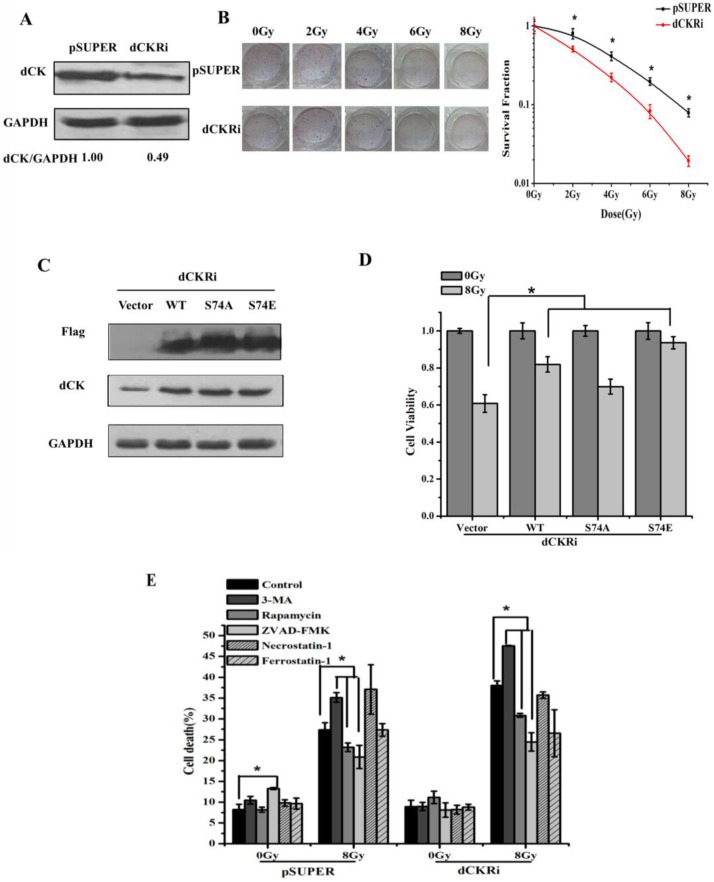
Deoxycytidine kinase (dCK) decreased radiation-induced cell death. (**A**) Establishment of dCK knock-down cells. Human cervical carcinoma cell line (HeLa) cells were stably transfected with pSUPER control or dCK shRNA. Individual clones were obtained under puromycin selection. Knock-down effects were confirmed by Western blot. Data were presented as mean ± SD of three independent experiments; (**B**) radiosensitivity was assessed by the colony formation assay in both dCK silencing cells and control cells after radiation treatment (0–8 Gy). Data were presented as mean ± SD of three independent experiments. * *p* < 0.05 versus control group or dCK silencing group; (**C**) dCK knock-down HeLa cells were reintroduced with vector control, dCK wild-type, dCK S74A mutant or S74E mutant. Overexpression of different dCK genotypes were shown by Western blot in HeLa cells. Data were presented as mean ± SD of three independent experiments; (**D**) the cells with different dCK genotypes were treated with 8 Gy radiation. Cell viability was analyzed by CCK-8 assay. * *p* < 0.05 versus control group; (**E**) the pSUPER and dCK knock-down cell lines were pretreated with 3-MA (2 mM), rapamycin (200 nM), ZVAD-FMK (10 μM), Necrostatin-1 (10 μM) or Ferrostatin-1 (5 μM) for 1 h, respectively, followed by ionizing radiation (IR) (8 Gy). After 48 h, cells were stained with trypan blue and analyzed by flow cytometry assay. * *p* < 0.05 versus control group.

In order to elucidate the roles of dCK in radiation-induced cell death, we established stable HeLa models with dCK shRNA and control shRNA (Figure 1A). The colony formation assays demonstrated that dCK knock-down increased radiosensitivity (Figure 1B). We transfected either empty vector, wild-type dCK, dCK S74A or S74E plasmid into dCK knock-down cells (Figure 1C). The plasmid dCK S74A has a Serine 74 to alanine substitution, which abrogates phosphorylation, and dCK S74E has a Serine 74 to glutamic acid substitution, which mimics phosphorylation. After IR treatment, a CCK-8 assay was conducted to assess cell viability (Figure 1D). We found that IR-induced cell death can be reversed by reintroduction of dCK, especially by dCK S74E, which improved survival by 84%, suggesting that dCK has positive effects on radioresistance. To further elucidate the types of cell death occurring, different regulators were used: we inhibited autophagy, apoptosis, necrosis and ferroptosis with 3-methyladenine (3-MA), ZVAD-FMK, Necrostatin-1, Ferrostatin-1, respectively, and induced autophagy by using Rapamycin. Necrostatin-1 and Ferrostatin-1 had no effect on IR-induced cell death, while Rapamycin and ZVAD-FMK decreased the rate of cell death by 19% and 36%, and 3-MA increased the rate of cell death by 25% in dCK knock-down cells, indicating apoptosis contributed to the majority of cell death (Figure 1E).

### 2.2. dCK Suppressed the Ionizing Radiation (IR)-Induced Apoptosis

**Figure 2:**
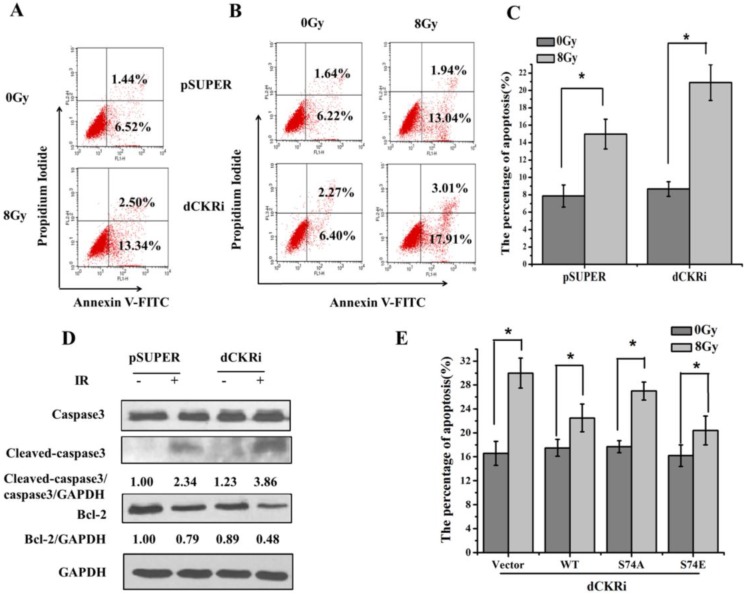
dCK silencing promoted IR-induced apoptosis. (**A**) Flow cytometry was used to quantify apoptosis in HeLa cells 24 h after radiation. Cells were stained with propidium iodide (PI) and Annexin V-FITC. The positive-stained cells were counted using FACScan; (**B**) apoptosis was detected in both control and dCK knock-down cell lines 24 h after radiation; (**C**) quantitative analysis of (**B**), data were presented as mean ± SD of three independent experiments. * *p* < 0.05 versus mock group; (**D**) whole-cell lysates were harvested and subjected to Western blot using the indicated antibodies; (**E**) dCK knock-down HeLa cells were reintroduced with vector control, dCK wild-type, dCK-S74A mutation or dCK-S74E mutation and then treated with IR (8 Gy). After 24 h, apoptotic rate was quantified by flow cytometry. * *p* < 0.05 versus mock group.

To confirm dCK contributed to IR-induced apoptosis, we tested IR-induced apoptosis in HeLa cells (Figure 2A). The flow cytometry assay showed that dCK participated in the regulation of apoptosis (Figure 2B,C). After IR treatment, a significant increase in apoptosis (141%) was found in the dCK knock-down cells as compared with pSUPER cells (91%). Western blotting showed that in dCK knock-down cells, IR induced more cleaved-caspase3 and less Bcl-2 expression as compared with the control group (Figure 2D), suggesting that dCK contributes to the IR-induced apoptosis. We then reintroduced dCK constructs to establish cell models with different dCK genotypes. After 8 Gy irradiation, apoptosis increased by 88% in vector cells, and increased by 50% in dCK-S74A cells. However, apoptosis showed only smaller increases of 29% and 26% in dCK-WT and dCK-S74E cells, suggesting phosphorylated dCK suppresses apoptosis induced by IR (Figure 2E).

### 2.3. dCK Promoted the IR-Induced Autophagy

**Figure 3:**
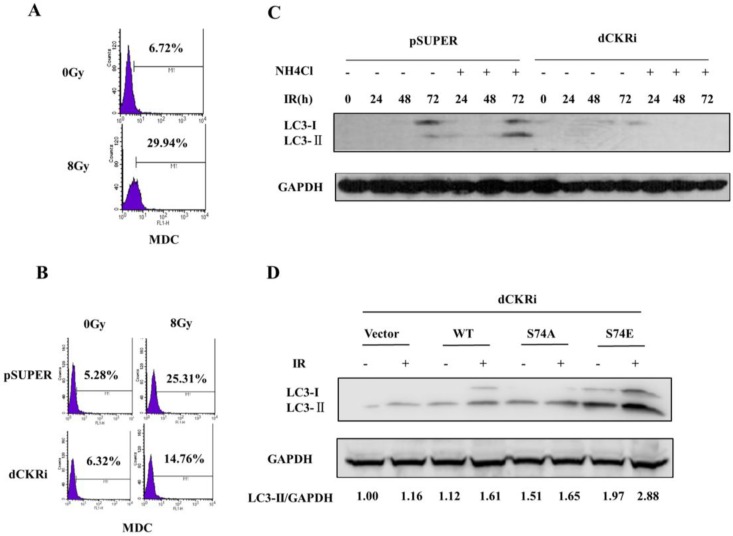
dCK promoted IR-induced autophagy in HeLa cells. (**A**) HeLa cells were treated with mock or IR (8 Gy). Staining of monodansylcadaverine (MDC) was detected by flow cytometry at 24 h after IR treatment; M1 represents fluorescent densities of MDC positive cells; (**B**) both dCK silencing cells and pSUPER cells were treated with 8 Gy radiation; 24 h later, staining of MDC was detected by flow cytometry; M1 represents fluorescent densities of MDC positive cells; (**C**) western blot analysis of microtubule-associated protein 1A/1B-light chain 3 (LC3) expression in the pSUPER and dCK knock down cells 24, 48 and 72 h after 8 Gy radiation. Glyceraldehyde-3-Phosphate Dehydrogenase (GAPDH) was used as an internal standard; M1 represents fluorescent densities of MDC positive cells; (**D**) dCK knock-down HeLa cells were reintroduced with vector control, dCK wild-type, dCK-S74A mutation and dCK-S74E mutation. 36 h after transfection, cells were exposed to mock or IR (8 Gy). 24 h after IR, cells were harvested and subjected to western blot using the indicated antibodies.

Since autophagy inhibitor 3-MA significantly increased IR-induced cell death (Figure 1E), we decided to test whether dCK participates in the regulation of radiation-induced autophagy. Flow cytometry was used to test the IR-induced autophagic rate (Figure 3A). It showed IR induced autophagy in HeLa cells. Besides that, autophagy induced by IR increased by 397% in pSUPER cells, and only by 134% in dCK knock-down cells, suggesting that dCK could increase IR-induced autophagy (Figure 3B). Ammonium chloride (NH4Cl) is a lysosomal inhibitor which can block organelle acidification and enable assessment of autophagic flux [27]. Western blotting revealed that LC3-II increased in a time-dependent manner (Figure 3C), reaching a peak in 72 h with NH4Cl+IR treatment in pSUPER cells, but there was low expression of LC3-II in dCK knock-down cells. In order to investigate whether dCK S74 phosphorylation is associated with IR-induced autophagy, we introduced dCK constructs into the dCK knock-down cells. Western blotting showed that after IR treatment, LC3-II protein levels increased by 44% in dCK-WT cells and by 46% in dCK-S74E cells, but only increased by 9% in dCK-S74A cells and by 16% in the control cells (Figure 3D), indicating that dCK S74 phosphorylation is involved in IR-induced autophagy.

### 2.4. Suppressing Autophagy Could Increase Apoptosis Induced by ionizing Radiation

**Figure 4:**
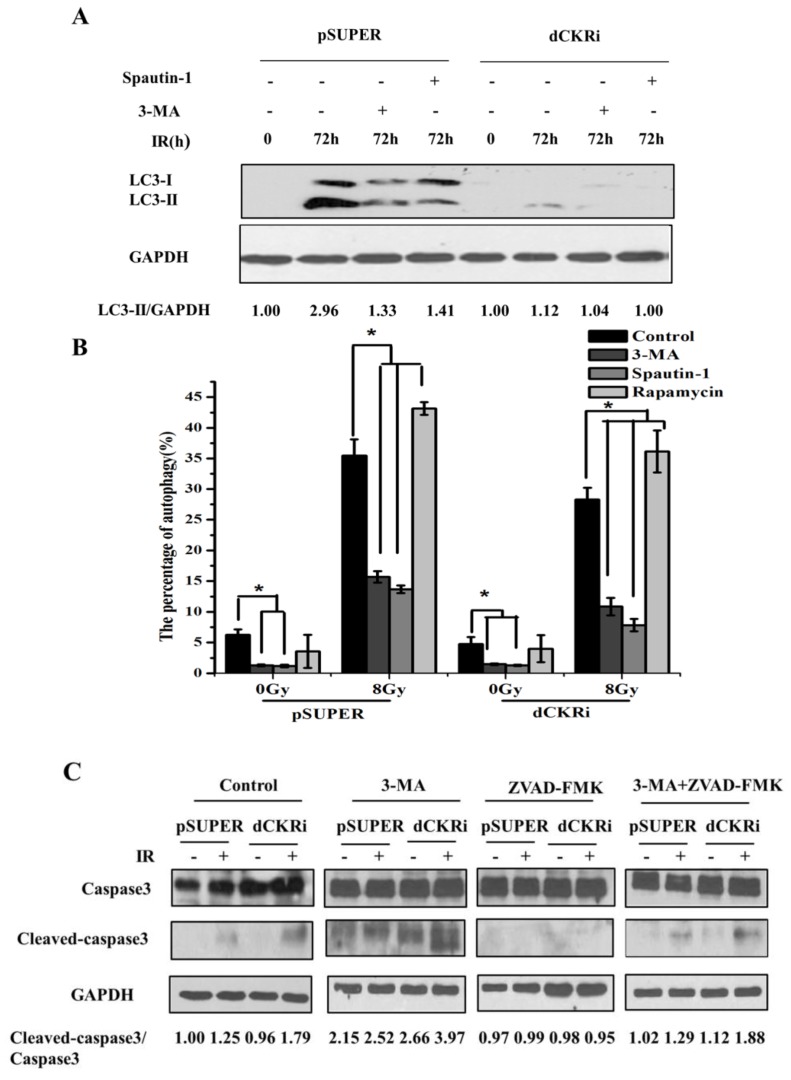

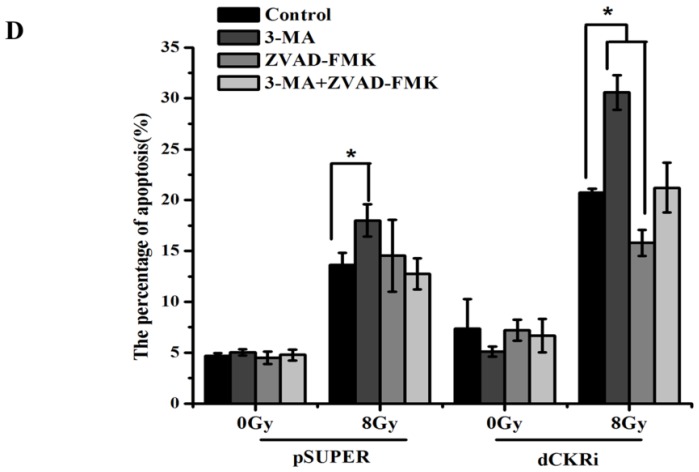
Suppressing autophagy could increase apoptosis induced by IR. (**A**) The pSUPER and dCK knock-down cell lines were incubated with spautin-1 (10 μM) or 3-MA (2 mM) for 1 h, and then treated with IR (8 Gy) for 72 h. Western blot analysis was performed to detect MAPLC3-I/II and GAPDH was used as an internal standard; (**B**) the control and dCK knock-down cells were pretreated with 3-MA (2 mM), spautin-1 (10 μM) or rapamycin (200 nM) for 1 h, and then treated with IR (8 Gy). After 24 h, staining of MDC was detected by flow cytometry. Quantitative analysis of autophagic cells was presented as mean ± SD of three independent experiments. * *p* < 0.05 versus mock group; (**C**) the pSUPER and dCK knock-down cell lines were incubated with 3-MA (2 mM), ZVAD-FMK (20 μM) or ZVAD-FMK (20 μM) + 3-MA (2 mM) for 1 h, then treated with IR (8 Gy) and incubated for 24 h. Western blot analysis was performed to detect caspase3 and cleaved-caspase3; GAPDH was used as an internal standard; (**D**) the pSUPER and dCK knock-down cell lines were pretreated with 3-MA (2 mM), ZVAD-FMK (10 μM), 3-MA (2 mM) + ZVAD-FMK (10 μM) for 1 h respectively, and then treated with IR (8 Gy). After 48 h, cells were collected, stained with PI and Annexin V-FITC and analyzed by flow cytometry. * *p* < 0.05 versus control group.

To further study the correlation between IR-induced autophagy and apoptosis, the autophagy inhibitors 3-MA and spautin-1 were pretreated for 1 h following IR treatment in pSUPER and dCK knock-down cells. (Figure 4A). The result showed that IR increased LC3-II protein by 196% in pSUPER cells and decreased by 55% and 52% in the presence of 3-MA and spautin-1, respectively. In dCK knock-down cells, IR treatment increased LC3-II protein by 12% and decreased LC3-II protein by 7% and 11% in the presence of 3-MA and spautin-1. This suggested that 3-MA and spautin-1 can inhibit IR-induced autophagy. We then used different autophagy regulators to check the change in autophagy flux by flow cytometry (Figure 4B).

Interestingly, 3-MA and spautin-1 inhibited IR-induced autophagy and rapamycin increased it in both cell lines. After radiation, LC3-II significantly decreased in dCK knock-down cells as Western blot shows (Figure 4A), while MDC staining only shows a slight decrease of autophagy in irradiated cells as compared with pSUPER. It might because the different efficacies of the two methods and Western blot is more sensitive than MDC for the detection of autophagy. Given that dCK plays an important role in the induction of both apoptosis and autophagy, we focused on the potential relationship between apoptosis and autophagy. Both 3-MA and ZVAD-FMK were used to inhibit autophagy and apoptosis, respectively. As shown in Figure 4C, IR increased cleaved-caspase3 protein by 86% in dCK knock-down cells but only by 25% in control cells. When cells were treated with ZVAD-FMK, cleaved-caspase3/caspase3 decreased in both cell lines. Interestingly, 3-MA pre-treatment caused a notable cleaved-caspase3/caspase3 increase in control and dCK knock-down cells after IR treatment. Furthermore, the ratio of cleaved-caspase3/caspase3 was much lower in the 3-MA + ZVAD-FMK-treated cells than in the 3-MA-treated cells. This suggests that autophagy inhibitor 3-MA may promote apoptosis. IR-induced apoptosis decreased more significantly in dCK knock-down cells (24%) with ZVAD-FMK treatment, and 3-MA increased IR-induced apoptosis by 48%. However, in control cells, 3-MA increased IR-induced apoptosis by 32%, while ZVAD-FMK did not significantly alter apoptosis induced by IR. Moreover, compared with the control group, there was no significant change of cell apoptosis in the 3-MA + ZVAD-FMK group in both cell lines (Figure 4D).

### 2.5. dCK Regulated IR-Induced Autophagy through mTOR Pathway

**Figure 5:**
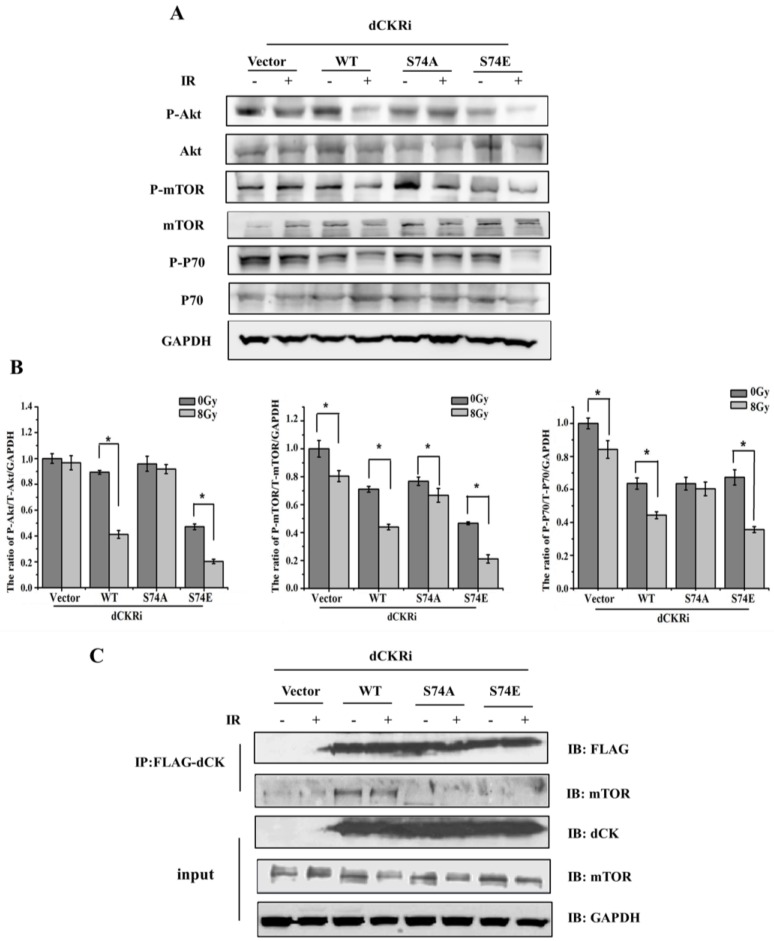
dCK Serine 74 phosphorylation participated in the regulation of autophagy induced by IR. (**A**) The dCK-complemented cell lines were treated with mock or IR (8 Gy). A total of 24 h after IR, cells were harvested and subjected to Western blot using the indicated antibodies; (**B**) quantitative analysis of (**A**), cells transfected with pSUPER and treated with mock were used as control (100%). Data were presented as mean ± SD of three independent experiments. * *p* < 0.05 versus cells treated with IR only; (**C**) vector control, FLAG-tagged wild-type or S74A dCK were transiently transfected into HeLa cells in which dCK was knocked-down. After 36 h of transfection, the cells were treated with mock or IR (8 Gy). Then, 24 h after IR, cells were harvested and subjected to co-immunoprecipitation using an anti-FLAG antibody. The co-immunoprecipitates were then blotted with indicated antibodies.

Cells under stress try first to survive by autophagy and if they notice that it is not possible, they die trough apoptosis. We have demonstrated that activated dCK by IR can increase autophagy (protect cells) and suppress apoptosis. Moreover, suppressing autophagy could increase apoptosis induced by IR. We next want to understand the specific mechanism of autophagy regulated by dCK. It is well known that mTOR serves as the negative regulator of autophagy [3]. In our study, Western blot analysis showed that IR treatment significantly decreased levels of phospho-Akt/Akt/GAPDH by 54%, phospho-mTOR/mTOR/GAPDH by 39% and phospho-P70S6K/P70S6K/GAPDH by 31% in the dCK-WT cells (Figure 5A,B). Moreover, IR treatment significantly decreased levels of phospho-Akt/Akt/GAPDH by 57%, phospho-mTOR/mTOR/GAPDH by 55% and phospho-P70S6K/P70S6K/GAPDH by 46% in dCK-S74E cells. However, the levels of phospho-Akt/Akt/GAPDH, phospho-mTOR/mTOR/GAPDH, phospho-P70S6K/P70S6K/GAPDH showed only smaller decreases of 8%, 13% and 5%, respectively, in dCK-S74A cells following IR treatment. This suggests that activated dCK could inhibit the Akt/mTOR/P70S6K pathway and promote autophagy with IR treatment. In order to understand how dCK participates in the Akt/mTOR/P70S6K pathway, we conducted a co-localization assay with specific antibodies. The result showed that dCK was co-localized with mTOR, which did not change significantly after IR treatment (Figure S1), i.e., the co-immunoprecipitation experiment showed that both inactivated (no IR) and activated (IR) dCK can interact with mTOR despite S74 mutants not being able to interact with mTOR (Figure 5C). Together, these results indicate that activated dCK promotes autophagy via the Akt/mTOR/P70S6K pathway in response to IR.

### 2.6. dCK Is Required for the G2/M Cell Cycle Checkpoint and to Protect Cells against Mitotic Catastrophe

**Figure 6:**
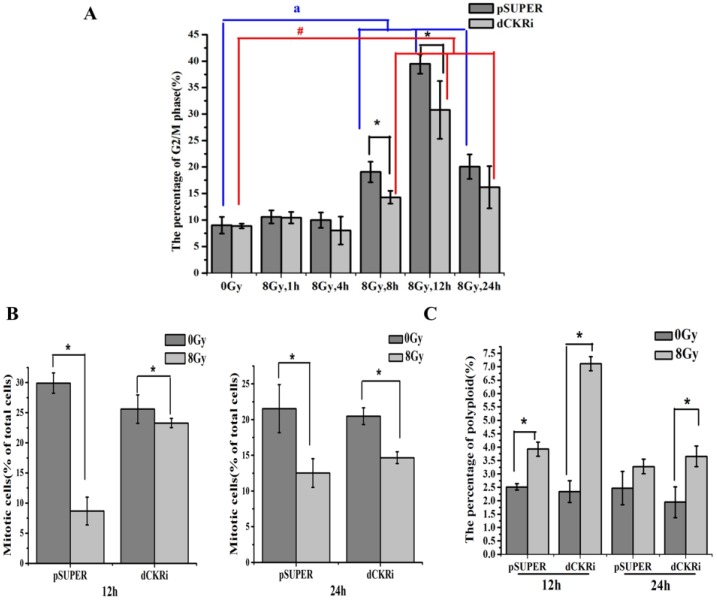
dCK inhibited IR-induced mitotic catastrophe. (**A**) Flow cytometry was used to quantitate the cell G2/M phase with or without IR at 1, 4, 8, 12, and 24 h in isogenic HeLa cells stably expressing control or dCK shRNA. All data are representative of three independent experiments and are shown as the mean ± SD. a *p* < 0.05 versus pSUPER cells treated with IR only, # *p* < 0.05 versus dCK knock-down cells treated with IR only. * *p* < 0.05 versus control group; blue lines are used to compare pSUPER cells treated with or without IR; red lineds are used to compare dCK knock-down cells treated with or without IR; (**B**) isogenic HeLa cell lines with control or dCK shRNA were treated with mock or IR treatment (8 Gy). 12 or 24 h later, cells were harvested and fixed followed by staining for Histone H3 Ser10 phosphorylation and analyzed by flow cytometry. The average and standard deviations of at least triplicate samples are shown. Statistic analysis was done by Student’s *t*-test; * *p* < 0.05 versus cells treated with IR only; (**C**) isogenic HeLa cell lines with control or dCK shRNA were treated with or without IR (8 Gy). After 12 or 24 h, cells were stained by DAPI and detected by flow cytometry. * *p* < 0.05 versus cells treated with IR only.

Our previous research has shown that dCK is required for G2 arrest [25]. In this study, we attempt to elucidate cell cycle changes that are associated with cell death. IR-induced G2/M phase arrest increased in a time-dependent manner (Figure 6A). Simultaneously, the G2/M phase arrest in dCK knock-down cells was lower than that in control cells after 12 h of IR treatment. In order to investigate whether dCK knock-down induced G2/M checkpoint abrogation, we tested mitotic cells by Histone H3 Ser10 phosphorylation staining. The result showed that the number of mitotic control cells decreased by about 70% 12 h after IR treatment. However, dCK knock-down had a minimal effect on mitotic cell number after 12 h IR treatment (Figure 6B). This suggests that dCK is required for activation of the IR-induced G2/M checkpoint. We know that mitotic catastrophe is associated with an abnormal cell cycle checkpoint, centrosomes, spindle and DNA damage. As such, we exposed control and dCK knock-down cells to 8 Gy irradiation and then stained with DAPI to identify mitotic catastrophe by flow cytometry (Figure 6C). IR increased polyploidy more significantly in dCK knock-down cells than in control cells (204% versus 56%) in the 12 h IR treatment group. This phenomenon was less obvious in the 24 h IR treatment group. Immediately after DNA damage, there is an early phosphorylation of Ser139 in histone at damage sites which appear quickly and disappear within a few hours [28,29]. Prolonged expression ofγH2AX suggests the abrogation of damage repair [30] and ERCC1 levels are associated with DNA repair capacity [31,32]. As Figure S2 shows, IR increased levels of H2AX and reduced ERCC1 considerably. ERCC1 expression was slightly lower in dCK knock-down cells as compared with parental cells, which indirectly supported the fact that loss of dCK increased the radiosensitivity by breaking the balance of DNA damage and repair. Overall, the results demonstrate that loss of dCK leads to abrogation of the G2/M checkpoint, thereby enabling abnormal cells to enter the mitosis phase, increasing DNA damage, reducing DNA repair and eventually inducing mitotic catastrophe in response to IR.

## 3. Discussion

It is known that many nucleoside analogs are used in combination with radiotherapy and deoxycytidine kinase (dCK) is required for the anti-tumor activity of these nucleoside analogs [21]. Moreover, dCK activation can be used as a biomarker for DNA damage response and Ataxia-Telangiectasia Mutated (ATM) function [24]. However, the roles of dCK in radiation-induced cell death are still unknown.

Given that dCK is activated in response to ionized radiation (IR) and that phosphorylation of dCK is crucial for its enzymatic activity [24,25], we introduced different dCK constructs into dCK knock-down cells. We found the dCK phosphor mimetic S74E was able to inhibit cell death induced by IR, while dCK S74A failed to do so, confirming that dCK S74 had an important role in cell death under IR treatment. We found that loss of dCK increased IR-induced cell death which was associated with autophagy and apoptosis, but was not associated with ferroptosis or necrosis.

Apoptosis is one of the most important mechanisms for cell death. dCK triggers the phosphorylation of cytarabine (Ara-C), CNDAC and other nucleoside analog drugs, which then inhibit tumor growth and promote apoptosis [33,34,35,36]. Hypoxia-induced dCK expression contributes to apoptosis [37,38]. Caspases are cysteine proteases that mediate apoptosis and Bcl-2 protein can inhibit apoptosis in vitro [39].To determine whether dCK is involved in IR-induced apoptosis, we detected caspase3 and Bcl-2 expression and discovered that, compared to the control cells, cleaved-caspase3 was increased and Bcl-2 expression was decreased in response to IR in dCK knock-down cells. Moreover, dCK (especially the S74E form) was able to decrease the rate of apoptosis induced by IR, suggesting dCK suppressed IR-induced apoptosis.

Autophagy is a lysosome-dependent self-digestion process [40] that promotes cell survival under some stresses, such as nutrient starvation, ROS, hypoxia, DNA damage and the unfolded protein response [41]. However when excessive cell damage is beyond the limit of repair under certain physiological conditions, autophagy turns into a programmed cell death mechanism (type II) [42]. We provided evidence that autophagy increased noticeably in response to IR in HeLa cells. We confirmed that dCK could increase LC3-II expression in a time-dependent manner in response to IR. It has been reported that dCK was activated in vivo by phosphorylation of Ser-74 [43]. We then wondered whether Ser-74 could affect autophagy induced by IR. As expected, IR treatment increased LC3-II expression noticeably in wild-type dCK and dCK-S74E cells, but not in S74A cells and the control cells, indicating that dCK S74 phosphorylation promoted IR-induced autophagy.

Spautin-1 is a specific and potent autophagy inhibitor-1 that can inhibit USP10 and USP13, and promote the degradation of Beclin-1 [44]. As an autophagy inhibitor, 3-MA (3-methyladenine) can suppress class III PtdIns 3-kinase [45]. We found that dCK increased IR-induced autophagy and this can be inhibited by 3-MA and spautin-1. Rapamycin is TOR (TOR complex 1) inhibitor, which induces autophagy. TOR complex 2 is much less sensitive to inhibition by rapamycin [46]. We found that autophagy regulators could cause a corresponding change in the incidence of autophagy and that dCK could change autophagy flux induced by IR. By using 3-MA and ZVAD-FMK to inhibit autophagy or apoptosis and rapamycin to induce autophagy, our data indicated that autophagy inhibition leads to an increase in IR-induced apoptosis in both pSUPER and dCK knock-down cells, which was more significant in pSUPER cells, indicating that dCK promoted IR-induced autophagy which protected cells from apoptosis induced by IR.

Given that dCK was required for autophagy induced by IR, we further investigated the mechanism by which dCK regulates IR-induced autophagy. Several signaling pathways such as mTOR, PI3K, Akt, Beclin-1 and HIF-1 have been reported to be involved in the regulation of autophagy [47]. dCK could regulate the migration and invasion through the AKT pathway and seems to be the upstream of AKT [48].We hypothesized that dCK-S74 phosphorylation might participate in the autophagy progress and found that the Akt/mTOR/P70S6K pathway could be suppressed obviously by IR in wild-type dCK and dCK-S74E cells. However, dCK-S74A did not change the Akt/mTOR/P70S6K pathway obviously in response to IR. Additionally, we presented data that wild-type dCK interacted with mTOR and the interaction did not change obviously after IR treatment. More interestingly, the S74A or S74E mutant dCK failed to co-immunoprecipitate with mTOR. We checked dCK sequence and found that dCK contained the LIR motif (LC3-interacting region) which was crucial for selective autophagy. If LIR location is close to S74, dCK S74E might be able to recruit LC3 to the LIR motif and S74A could also change the spatial conformation of dCK, consequently interfering with the interaction between dCK and mTOR. These results indicate that activated dCK may inhibit the Akt/mTOR/P70S6K pathway via interaction with mTOR and that dCK S74 participates in regulation of autophagy initiation with IR treatment.

Our previous studies have demonstrated that dCK functions as a critical regulator of the G2/M checkpoint. Cyclin-dependent kinase 1 (Cdk1) is associated with dCK in response to DNA damage. dCK can interact with Cdk1 after IR and the interaction inhibits Cdk1 activity [25]. Data presented in this manuscript confirmed that IR could induce G2/M phase arrest; however, silencing dCK resulted in a G2/M checkpoint defect, suggesting that dCK is required for activation of the IR-induced G2/M checkpoint. Besides that, dCK decreased the number of mitotic cells in response to IR, especially after 12 h IR. We know that when DNA is damaged, cells cannot divide completely, leading to significant increases in polyploidy levels and eventually causing mitotic catastrophe [49]. Our flow cytometry assay showed that IR-induced polyploidy increased significantly in dCK knock-down cells, especially in the 12 h IR treatment group. Moreover, loss of dCK suppressed DNA repair. As such, dCK has an important role in protecting cells from IR-induced mitotic catastrophe.

## 4. Materials and Methods

### 4.1. Cell Line, Antibody and Reagents

Human cervical carcinoma cell line, HeLa, was cultured in Dulbecco’s Modified Eagle Medium (DMEM) (GIBCO, Carlsbad, CA, USA) supplemented with 10% fetal bovine serum (FBS) and 1% penicillin/streptomycin (Invitrogen, Carlsbad, CA, USA) in glass Petri dishes at 37 °C in a 5% CO_2_ incubator.

Anti-dCK antibody was purchased from Abcam Inc (Cambridge, MA, USA). Anti-MAPLC3, anti-P-mTOR, anti-P-Akt, anti-P-P70S6K, anti-caspase3, anti-Bcl-2, anti-H2AX and anti-ERCC1 antibodies were purchased from Cell Signaling. Anti-GAPDH was obtained from Santa Cruz Biotechnology (Santa Cruz, CA, USA). The peroxidase-conjugated anti-mouse IgG and peroxidase-conjugated anti-rabbit IgG were purchased at Santa Cruz. Fetal bovine serum (FBS), and Cell Counting Kit-8 (CCK-8) was purchased from Dojin Laboratories (Kumamoto, Japan), 3-Methyladenine (3-MA), NH4Cl, Necrostatin-1, Ferrostatin-1, Rapamycin, Spautin-1 and monodansylcadaverine (MDC) were purchased from Sigma Chemical (St. Louis, MO, USA), ZVAD-FMK was purchased from Selleckchem (Houston, TX, USA), and pSUPER retroviral vector was obtained from OligoEngine (Seattle, WA, USA).

### 4.2. Radiation

X-ray generator (X-RAD 320ix, Precision X-ray Inc., North Branford, CT, USA) was utilized to deliver radiation at a dose rate of 1.0 Gy/min.

### 4.3. Plasmids

Wild-type dCK, dCK-S74A, dCK-S74E mutants were gifts from Bo XU (Southern Research Institute, Birmingham, AL, USA). ShRNAs were designed according to “www.idtdna.com.” ShRNAs were synthesized, denatured, ligated to pSUPER vector at BglII and HindIII sites. The primers used are shown as follows: dCK-WT-F 5′-tcactagtatggccaccccgcccaagagaagc-3′; dCK-WT-R 5′-acgctcgatcacaaagatctcaaaaactctt-3′; dCK-S74A-F 5′-cttacaatggcacagaaaaatggtgg-3′; dCK-S74A-R 5′-ccaccatttttctgtgccattgtaag-3′; dCK-S74E-F 5′-cttacaatggaacagaaaaatggtgg-3′; dCK-S74E-R 5′-ccaccatttttctgttccattgtaag-3′; dCK-shRNAs-F 5′-gatctgtggttcctgaacctgttgttcaagagacaacaggttcaggaaccacttttta-3′; dCK-shRNAs-R 5′-agcttaaaaagtggttcctgaacctgttgtctcttgaacaacaggttcaggaaccaca-3′.

### 4.4. Establishment of Deoxycytidine Kinase (dCK) Silencing Cells

The pSUPER-dCK and the vector with a scrambled sequence, i.e., pSUPER, were constructed in our lab. Cells were transfected with shRNA vectors to establish the dCK knock-down genotype. Both ShRNA vectors (10 μg) and package vector, Amphopack, were transfected into 293T, then the supernatant containing a pseudovirus particle was retrieved 72 h post-transfection and used to infect HeLa cells. Positive stable clones were selected by growing cells with puromycin (0.8 μg/mL) for 1 week.

### 4.5. Western Blot Analysis

Cells were harvested and the total proteins were extracted with RIPA (radioimmunoprecipitation) lysis buffer (NaCl (50 mM), HEPES, EGTA (2.5 mM), EDTA (1 mM), dithiothreitol (DTT, 1 mM), NP-40 (1%), NaF (10 mM), SV (1 mM), SDS (0.1%), PMSF (1 mM)), and 1 mL aliquot was mixed with 10 μL protease inhibitor cocktail. Identical amounts (50 μg/lane) of protein samples were subjected to electrophoresis on 8%–15% Tris-glycine SDS polyacrylamide gel, subsequently transferred to nitrocellulose. Membranes were then blocked with 5% skimmed milk in PBS at room for 1 h, incubated with primary antibodies followed by horseradish peroxidase-conjugated secondary antibodies, and visualized with the chemiluminescence system (Santa Cruz, CA, USA). The intensities of protein bands were quantified using image software (Quantity One, Guangzhou, China).

### 4.6. Co-Immunoprecipitation

Cells were harvested and lysed using RIPA buffer. Whole-cell lysates were incubated with Anti-FLAG-M2 Agarose beads (Sigma, St. Louis, MO, USA) overnight at 4 °C. The samples were then washed with TBST three times, eluted in 2× sample buffer and placed in a boiling water bath for 5 min. Western blot was used to analyze the eluted proteins.

### 4.7. Immunofluorescence Microscopy Analysis

There were 8 × 10^4^ cells seeded into 60 mm Petri dishes using standard culture medium and incubated for 24 h. A total of 24 h after different treatments, cells were washed three times with PBS, and fixed with methanol for 30 min. After washing with PBS, cells were incubated with primary antibodies at 4 °C for one night. Then cells were washed with PBS and stained with a secondary antibody for 1 h. After washing with PBS three times, cells were stained with DAPI for 15 min. Cell images were captured with Leica microsystems.

### 4.8. Colony Formation Assay

Cells were seeded into six-well plates in triplicate using standard culture medium. After 24 h, cells were irradiated (0, 2, 4, 6 and 8 Gy). Two weeks later, cells were fixed with 4% formaldehyde, and stained with 0.5% crystal violet. Then the number of colonies (more than 50 cells) was counted and normalized to the corresponding non-irradiated control. Cell survival curves were made by the multitarget click model of GraphPad Prism 5.0 (Systat Software, San Jose, CA, USA).

### 4.9. Cell Counting Kit-8 (CCK-8) Assay

Radiosensitivity was detected by Cell Counting Kit-8 assay (CCK-8, Dojin Laboratories, Kumamoto, Japan). There were 1 × 10^4^ cells/well seeded in 96-well plates containing complete medium. After 48 h, following different treatments, cells were incubated with CCK-8 solution (10 μL/well) for 2 h in an incubator. Microplate reader (Synergy HT, Bio-Tek, Winooski, VT, USA) was used to measure the absorbance at 450 nm.

### 4.10. Flow Cytometry Analysis

Cells were plated into six-well plates and treated with IR; cells were then collected 24 h after IR. For cell cycle analysis, cells were stained with RNase-containing PI (propidium iodide) solution. For polyploidy detection, cells were stained with DAPI. For autophagy detection, cells were stained with MDC. For apoptosis detection, cells were stained with PI and FITC-labeled Annexin-V. For cell death analysis, cells were stained with trypan blue. Stained cells were detected by Flow Cytometry (BD Biosystems, San Jose, CA, USA), data were analyzed with CellQuest (BD Biosciences, San Jose, CA, USA) and FlowJo softwares (Tree Star Inc., Ashland, OR, USA).

### 4.11. Statistical Analysis

All the data for the statistical analysis are obtained from at least three independent experiments. Statistical evaluations are presented as mean ± SD. The significance of the differences between groups was determined by one-way ANOVA; *p* < 0.05 was considered significant.

## 5. Conclusions

In summary, our data demonstrated that dCK could inhibit IR-induced cell death through suppression of apoptosis and mitotic catastrophe and promotion of autophagy. dCK increased autophagy induced by IR via inhibition of the Akt/mTOR/p70 S6K pathway and dCK S74 phosphorylation participated in this regulation. We demonstrated that dCK interacted with mTOR while dCK-S74A failed to do so. Furthermore, dCK was required for the G2/M checkpoint. Loss of dCK inactivated the G2/M checkpoint, allowing cells less time for repair and promoting entry into mitosis, inducing polyploidy increase, and eventually inducing mitotic catastrophe in response to IR.

## Supplementary Materials

Supplementary materials can be found at www.mdpi.com/1422-0067/17/11/1939/s1.
