# Supplementary material for: The Role of Deoxycytidine Kinase (dCK) in Radiation-Induced Cell Death
Source: Int J Mol Sci. 2016 Nov 21;17(11):1939. doi: 10.3390/ijms17111939 (PMC5133934; doi:10.3390/ijms17111939)
Supplement: Supplementary file 1 [file ijms-17-01939-s001.pdf]

Supplementary Materials: The Role of dCK in Radiation-Induced Cell Death

Rui Zhong, Rui Xin, Zongyan Chen, Nan Liang, Yang Liu, Shumei Ma and Xiaodong Liu

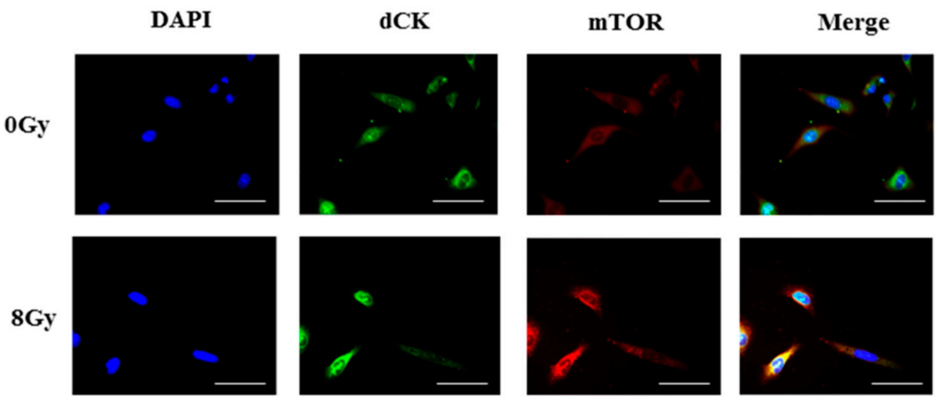

Figure S1. dCK was co-localized with mTOR. Cells were fixed and then immunostained by DAPI (blue), dCK antibody (green) and mTOR antibody (red). Scale bar, 10 μm.

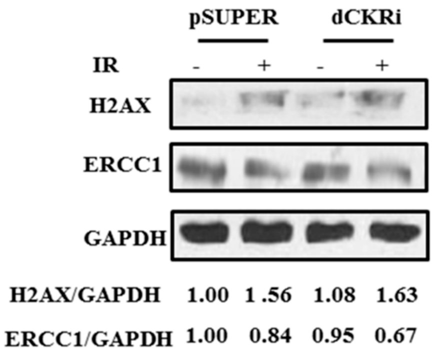

Figure S2. Expression levels of H2AX and ERCC1 following IR treatment. Isogenic HeLa cells stably expressing control or dCK shRNA were treated with mock or IR (8 Gy). 12 h after radiation, whole cell lysates were harvested and subjected to western blot using indicated antibodies.
